# Case Report: Congenital absence of the fifth metacarpal with polydactyly and syndactyly

**DOI:** 10.3389/fped.2025.1631029

**Published:** 2025-08-20

**Authors:** Zhihong Qin, Xiaolin Luo, Xiaofei Ding, Shijie Liao

**Affiliations:** The First Affiliated Hospital of Guangxi Medical University, Nanning, China

**Keywords:** congenital metacarpal agenesis, polydactyly, syndactyly, surgery, case

## Abstract

Congenital hand malformations encompass various types, with polydactyly and syndactyly being common. However, congenital absence of the metacarpal is rarely reported, and literature on this condition remains limited. To date, no cases of congenital absence of the fifth metacarpal combined with polydactyly and syndactyly have been documented. Here, we present a case of a 7-month-old female infant with congenital absence of the fifth metacarpal accompanied by polydactyly and syndactyly. The intraoperative management strategy is described, and early postoperative outcomes are evaluated. This case report aims to provide treatment approaches and clinical experience for complex congenital hand malformations, offering reference for clinicians managing similar cases in the future.

## 1 Introduction

The incidence of congenital upper limb and hand abnormalities is approximately 0.25% (1, 2). Among these, polydactyly and syndactyly are clinically common (3), while congenital metacarpal agenesis is rare, typically presenting as cleft hand deformity. Research on congenital fifth metacarpal agenesis remains extremely limited; a literature review identifies only four relevant reports (Table 1) (4–7). Notably, complex hand malformations involving fifth metacarpal agenesis combined with polydactyly and syndactyly (without associated syndromes) have not been documented in the literature.

**Table 1:** List of related cases.

Patient	Hands	Other abnormalities	Treatment
1 (6)	Left	Congenital dislocation of the left radial head	Pollicization of the left index finger; left radial head excision
2 (6)	Left	Congenital dislocation of the left radial head; right thoracic scoliosis	Pollicization of the left index finger; web space deepening
3 (6)	Left	Right ulnar club hand with absent fourth and fifth rays	Conservative treatment
4 (6)	Left	None	Conservative treatment
5 (6)	Left	Right thoracic scoliosis	Left little-ring finger web space deepening
6 (6)	Bilateral	None	Conservative treatment
7 (6)	Left	–	Removal of little finger recommended
8 (7)	Bilateral	–	Conservative treatment
9 (5)	Left	None	Proximal phalangeal osteotomy
10 (4)	Right	None	Removal of little finger recommended

For congenital metacarpal absence, no classification systems or treatment guidelines exist, though thumb polydactyly classifications have been proposed (8). The Oberg-Manske-Tonkin (OMT) classification roughly categorizes congenital hand malformations (9, 10). Surgery for hand deformities is typicallyperformed at 6–18 months to restore partial function and improve aesthetics (11). Surgical approaches include skin incision techniques (12–15), bone reconstruction (15, 16), and muscle insertion repair (17), all aimed at enhancing finger function. Thus, hand surgeons must design personalized plans to minimize complications and optimize mobility/appearance.

This case report describes congenital absence of the fifth metacarpal with polydactyly and syndactyly, presenting surgical management, early follow-up outcomes, and a literature review. The study aims to: (1) assess the rationale and feasibility of surgical intervention; (2) evaluate early efficacy by comparing pre- and postoperative status; (3) explore the combination of fifth metacarpal absence with other malformations and derive treatment strategies from existing literature.

## 2 Case report

### 2.1 Preoperative data

#### 2.1.1 Clinical findings

A 7-month-old female infant was admitted for a 7-month history of left-hand deformity. She had no comorbidities or syndromic features; the right hand was normal. No family history of polydactyly or syndactyly was noted.

#### 2.1.2 Physical examination revealed

**Figure 1:**
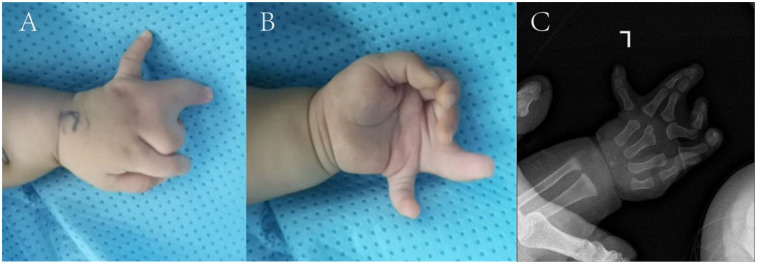
**(A,B)** Preoperative appearance and, **(C)** x-ray films.

Upon admission, the height was measured as 75 cm and the weight as 9 kg. left third finger with interphalangeal joint flexion deformity and poor flexion-extension function; syndactyly between the third and fourth proximal phalanges (web formation); complete syndactyly of the fourth and fifth fingers with flexed interphalangeal joints, absent active movement, and limited passive range of motion; a sixth finger with ∼75° ulnar deviation, active flexion-extension, and mild restriction (Figures 1A,B).

#### 2.1.3 Radiological features

Imaging revealed four metacarpals in the left hand, with the third metacarpal distal end hypertrophied. The left middle finger's proximal phalanx was hypertrophic, articulating with the third metacarpal and showing mild joint space widening. The left ring finger had two sets of phalanges (polydactyly changes); its radial supernumerary segment and the third metacarpal showed mild joint space widening. The ring finger's two distal phalanges were distally fused with trabecular perforation. The left index finger had distal interphalangeal joint flexion, and the middle finger had proximal interphalangeal joint flexion. Soft tissue syndactyly was noted between the left middle finger and proximal ring finger. The left little finger's proximal phalanx articulated with the ulnar aspect of the fourth metacarpal's distal end (Figure 1C). The ulna, radius, carpals, and remaining metacarpals were normal in shape and number.

#### 2.1.4 Surgical planning

Complete resection of the supernumerary syndactylous digits; correction of flexion deformity of the third finger; formation of a metacarpophalangeal joint between the third finger and third metacarpal (shared with the fourth finger); establishment of a metacarpophalangeal joint between the fifth finger and fourth metacarpal. Tendon preservation was prioritized to facilitate subsequent functional reconstruction of the fingers.

### 2.2 Main intraoperative conditions and treatment

#### 2.2.1 Management of polydactyly and syndactyly

First, syndactyly and polydactyly were addressed. A Z-plasty flap was designed on the radial side of the supernumerary fourth finger. After dissection and exposure, the supernumerary phalanx of the fourth finger was resected at the metacarpophalangeal joint level (with partial distal phalanx beneath the nail bed preserved). The extensor tendon was retained for extensor function reconstruction of the remaining ulnar finger (remaining fingers were labeled per normal sequence due to supernumerary digit excision).

#### 2.2.2 Management of the middle finger and ring finger

**Figure 2:**
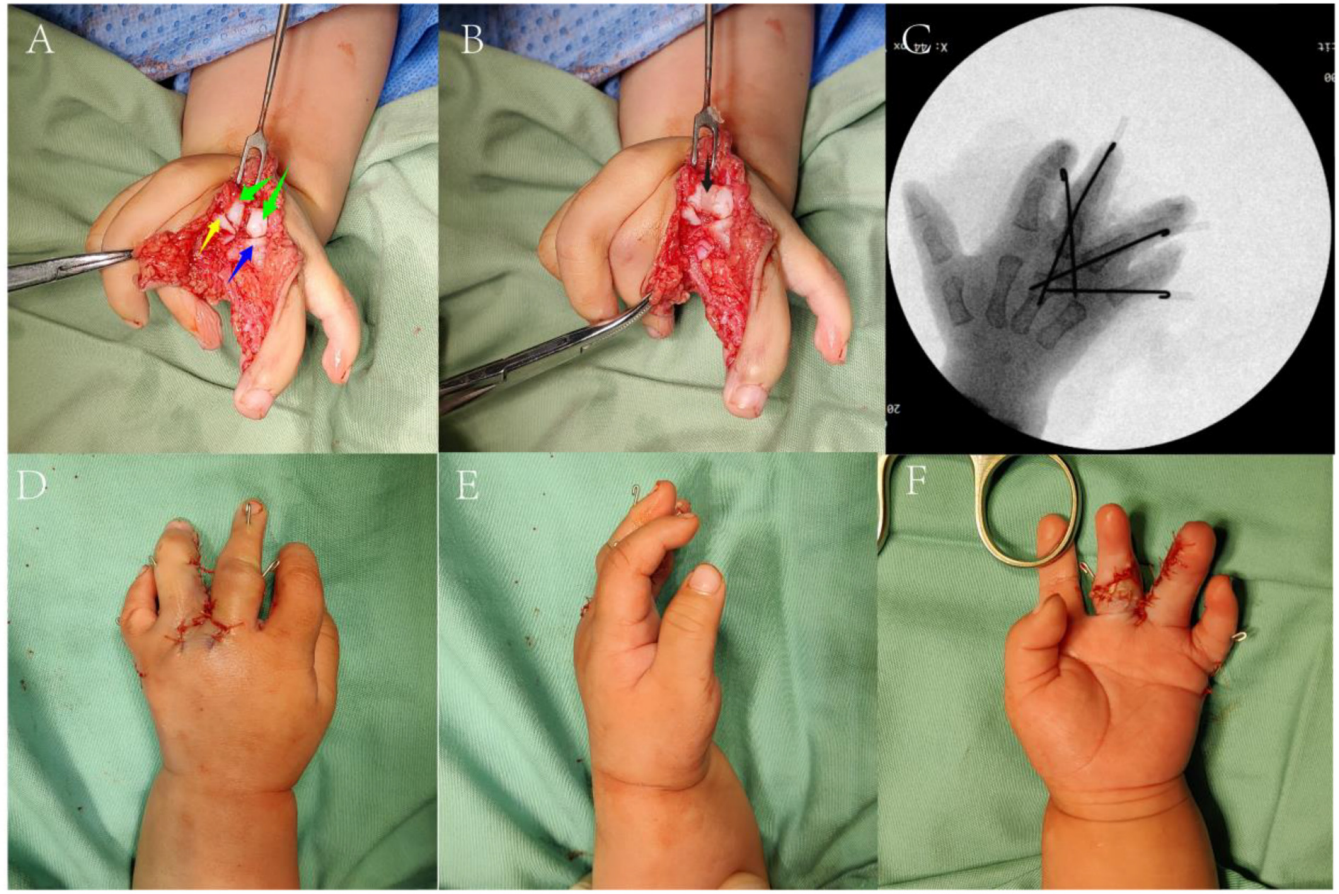
Intraoperative images **(A,B)** the main intraoperative procedures. (Green arrow: the separated articular surface of the third metacarpal head; yellow arrow|: proximal end of the third proximal phalanx; blue arrow: proximal end of the fourth proximal phalanx; black arrow: grafted bone wax). **(C)** Intraoperative fluoroscopy. **(D–F)** Appearance after suture during the operation.

A double Z-plasty flap was designed on the dorsal metacarpal region of the middle and ring fingers to reconstruct the web space. The flap was incised to expose the interphalangeal and metacarpophalangeal joints of these fingers; contractures of the ulnar collateral ligaments of the middle finger's interphalangeal and metacarpophalangeal joints were then released. The third metacarpal head was hypertrophic with two articular surfaces (wider radial surface) connected by cartilage. Gentle dissection separated the two surfaces, which were trimmed. Under protective dressing, the head was split into two segments: bone graft was placed beneath the articular cartilage (Figure 2A), and bone wax filled the gap between articular surfaces (Figure 2B) to reconstruct the third and fourth metacarpophalangeal joints. Remaining joint capsules of the middle and ring fingers were repaired and tightened to enhance interphalangeal stability; concurrent tightening of the radial collateral ligament of the interphalangeal joint preliminarily corrected the middle finger deformity. The ring finger's interphalangeal joint capsule was sutured, and the preserved extensor tendon was attached to the proximal middle phalanx of the ring finger for extensor function reconstruction.

#### 2.2.3 Management of the little finger

An incision on the ulnar side of the fifth finger allowed release of contracted collateral ligaments, correction of ulnar deviation, and repositioning at the fourth metacarpal level, enabling articulation between the fifth finger's proximal phalanx and fourth metacarpal to form a metacarpophalangeal joint. All joints were stabilized with Kirschner wires (Figure 2C), and surrounding tissues were sutured to reconstruct capsules. Post-reconstruction, finger morphology and alignment of the middle, ring, and little fingers were satisfactory. After thorough irrigation, the flap was trimmed to reconstruct the web space (Figures 2D–F), concluding the procedure.

### 2.3 Postoperative and follow-up

**Figure 3:**
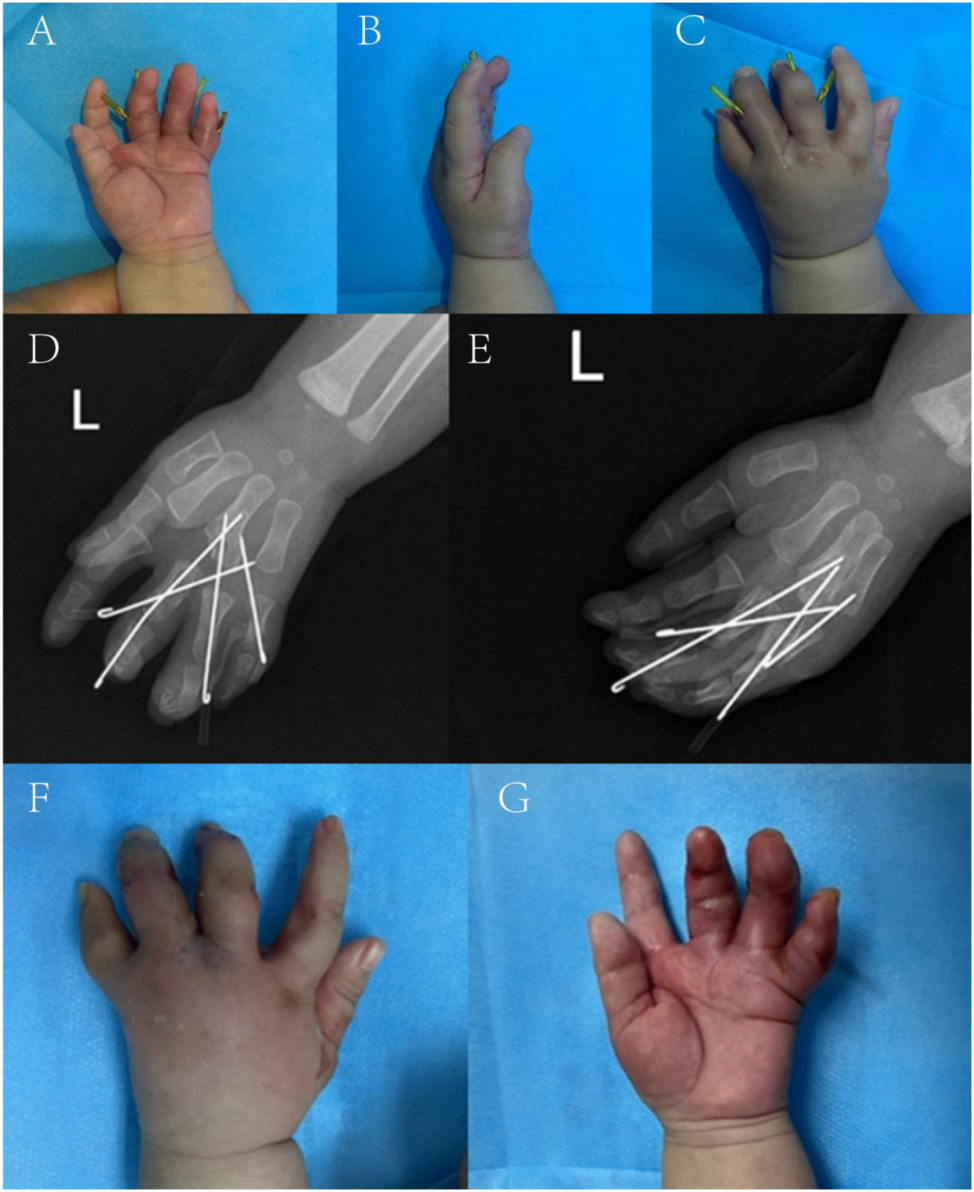
Follow-up pictures at five weeks after the operation. **(A–C)** Appearance photographs. **(D,E)** x-ray films. **(F,G)** Appearance after the removal of Kirschner wire.

At 5 weeks postoperatively, follow-up examination showed good perfusion of the web flap with no necrosis; the left hand was mildly swollen but with satisfactory cosmesis (Figures 3A–C). Left hand radiographs (Figures 3D,E) prompted Kirschner wire removal (Figures 3F,G), after which brace protection was continued. The parents were satisfied with the outcome.

**Figure 4:**
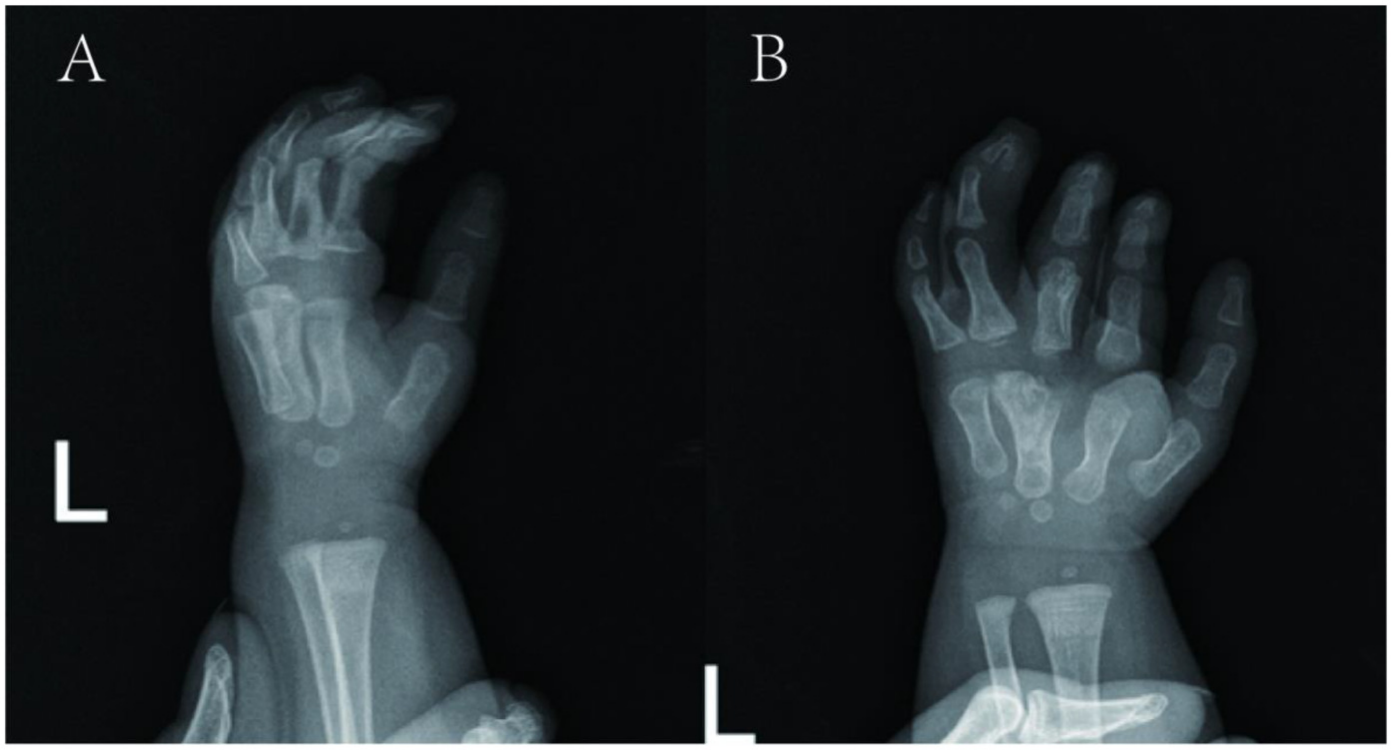
Follow-up pictures at ten weeks after the operation. **(A,B)** x-ray films.

At 10 weeks, a second follow-up revealed the x-ray findings in Figures 4A,B. Hand perfusion and sensation were intact, though little and ring finger functions remained incompletely recovered. The overall result was satisfactory to the family.

**Figure 5:**
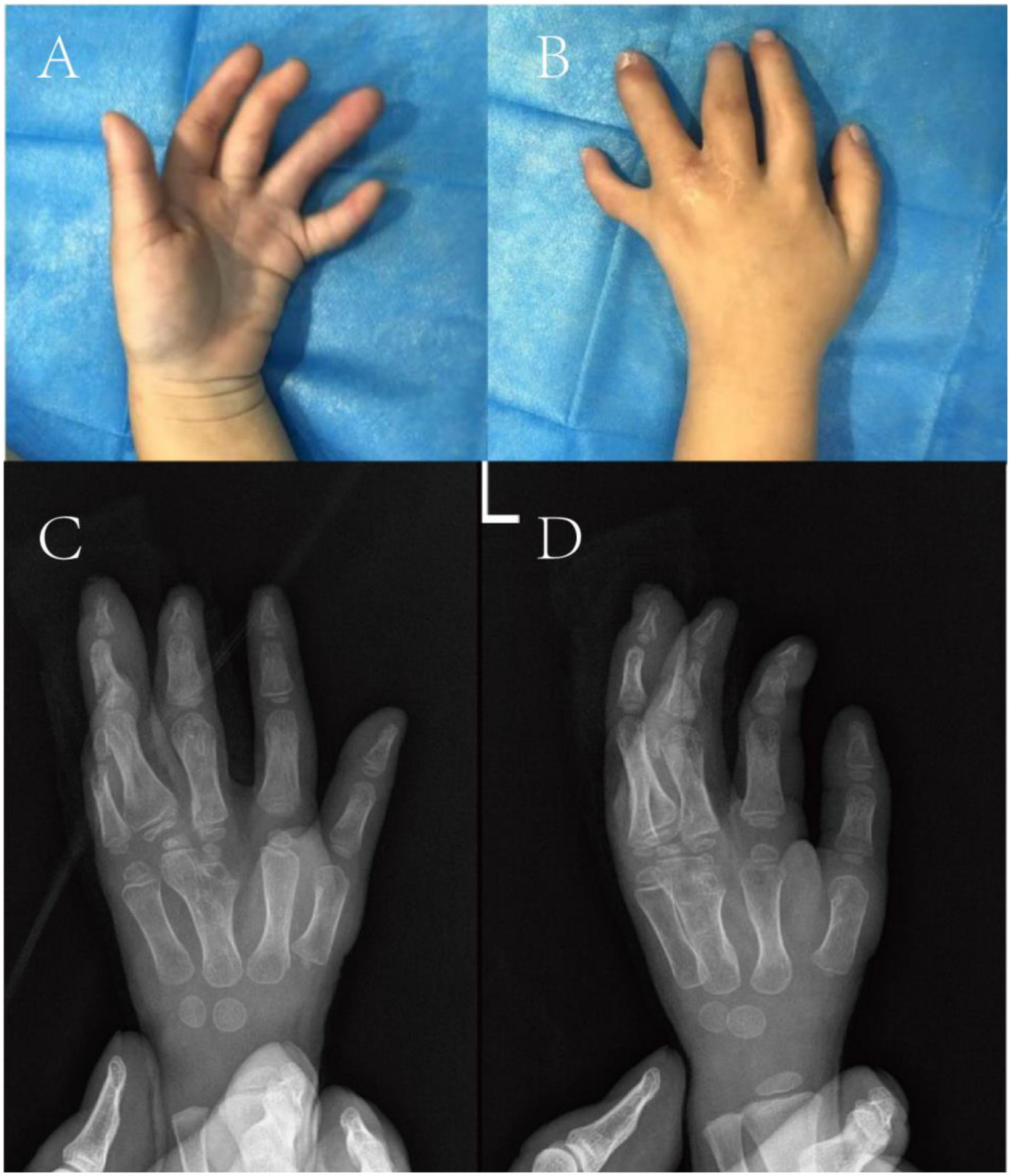
Follow-up pictures at one year after the operation. **(A,B)** Appearance photographs. **(C,D)** x-ray films.

At 1 year postoperatively, follow-up (Figures 5A,B) showed x-ray evidence of a distal fourth metacarpal epiphysis in the surgically separated Y-shaped reconstructed metacarpal (Figures 5C,D), which formed metacarpophalangeal joints with the proximal third and fourth phalanges, respectively, with satisfactory alignment. Gross examination revealed the fourth finger was larger and longer than the third, with a narrow web space between them. The fifth finger had ∼15° ulnar deviation at rest. Active movement showed: third finger metacarpophalangeal joint flexion to 10°, proximal interphalangeal joint to 75°, and distal interphalangeal joint to 50°; fourth and fifth fingers had stiff metacarpophalangeal and interphalangeal joints with only ∼10° active movement.

Hand appearance was significantly improved aesthetically vs. preoperative status. However, stiffness and poor function of the fourth and fifth finger joints persisted, warranting consideration of further surgery for functional enhancement.

## 3 Discussion

The optimal surgical timing for congenital hand malformations is generally 6–18 months (18, 19), with the primary goals of restoring partial hand function and achieving satisfactory aesthetics (20). Delayed surgery may exacerbate deformities during growth, impairing hand function and quality of life; aesthetic deficits can also impose social and psychological burdens (21). Thus, preoperative confirmation of diagnosis, associated abnormalities, and syndromic associations is critical. Inaccurate diagnosis or inadequate differential diagnosis may worsen outcomes (22).

For congenital fifth metacarpal agenesis with associated malformations, management strategies vary based on finger function. Buckwalter et al. (6) reported 7 cases of congenital fifth metacarpal agenesis, 4 of which were associated with other malformations. Among these 7 patients, 2 with thumb agenesis underwent index finger pollicization, 1 underwent web space deepening, 1 underwent little finger amputation, and the remaining 3 opted for conservative management. Eren F et al. (7) reported a case of bilateral congenital fifth metacarpal agenesis without associated malformations. The patient presented with normal hand function but sought medical attention due to cosmetic dissatisfaction, ultimately opting for conservative management. Peker F et al. (5) reported a case of congenital absence of the metacarpal, where the ring and little fingers shared the fourth metacarpal to form a common metacarpophalangeal joint. The left ring and little fingers showed reduced web space at the proximal interphalangeal joints, with hypoplasia and shortening compared to the contralateral side. Despite limited motion without significant dysfunction, the patient sought surgery for cosmetic concerns, undergoing proximal phalangeal wedge osteotomy of the little finger with mini-plate fixation of the osteotomy site. Barnett SA et al. (4) reported a case of congenital fifth metacarpal agenesis in the right hand, with the ring and little fingers sharing the fourth metacarpal to form a common metacarpophalangeal joint. The inter-digital web space between the ring and little fingers was shallow, and the little finger exhibited flexion deformity with hypoplasia; other fingers had normal function. The patient ultimately underwent little finger amputation.

In our reported case, unlike those in the previous literature review, congenital absence of the fifth metacarpal is accompanied by polydactyly and syndactyly, without other limb malformations or associated syndromes. Previous surgical interventions described in the literature have limited reference value for surgical management. We were inspired by the study by Bergmeister K et al. (23) in which the entire fifth metacarpal was resected due to metacarpal bone tumor. Subsequently, autologous iliac bone graft was harvested and fused with the fourth metacarpal, followed by fixation of the proximal end of the proximal phalanx of the little finger to the grafted iliac bone at a 30° flexion angle. Postoperative follow-up showed that the patient had good hand function, except for fusion at the metacarpophalangeal joint of the little finger. Thus, we divided the articular surface of the third metacarpal into two separate surfaces using bone wax, forming joints with the middle and ring fingers respectively. Preliminary follow-up results showed satisfactory outcomes, though long-term functional efficacy requires further observation. For joint ligament management, contracted-side ligaments were released while the contralateral ligaments were sutured; partial tendon preservation was performed to enhance functional mobility of the remaining fingers. Regarding the transposition of the ring finger to the third metacarpal and the little finger to the fourth metacarpal for metacarpophalangeal joint formation: preoperatively, the ring finger had minimal voluntary movement, whereas the little finger retained voluntary function. Our strategy prioritized preserving fingers with voluntary function to maximize residual hand function, a decision validated by initial follow-up. Additionally, the distal phalanx of the supernumerary digit was preserved to avoid nail bed injury and optimize the cosmetic appearance of the ring finger.

In previously reported cases, conservative management or web space deepening was preferred for patients with good function but cosmetic dissatisfaction. For those with hypoplastic little fingers and limited mobility, little finger amputation was an option.Given the greater rarity and complexity of our reported case compared to previous ones, more considerations were required to achieve both cosmetic improvement and functional recovery.

## 4 Study limitations

Given the paucity of reference cases, surgical planning was guided by principles for polydactyly and syndactyly in children. Intraoperatively, after resecting the original fourth finger, the third metacarpal head was split into two articular surfaces; the phalanges and metacarpals were then realigned and stabilized with Kirschner wires. Although a satisfactory cosmetic outcome was achieved postoperatively, no significant improvement was observed in the mobility of the 3rd to 5th fingers. Potential contributing factors include: 1. Incomplete matching of the metacarpophalangeal joints after realignment, resulting in restricted joint mobility; joint stiffness secondary to Kirschner wire fixation; 2. Incomplete tendon-bone healing following tendon preservation and insertion reconstruction; hypoplasia of digital tendons with insufficient flexion/extension strength; tendon adhesions; 3. Joint capsule contracture with restricted mobility; 4. Inadequate postoperative rehabilitation exercises. In contrast to the approach described by Bergmeister K et al. (23), we avoided direct metacarpophalangeal joint fusion in functional position, as our patient was a 7-month-old infant with growth potential.

## 5 Conclusion

We report a rare case of congenital fifth metacarpal agenesis combined with polydactyly and syndactyly. Preoperatively, the left hand presented with obvious deformity and restricted function. Following preoperative deliberation, the aforementioned surgical procedures were performed. Initial follow-up showed satisfactory outcomes, though long-term results require further follow-up and observation.

## Data Availability

The original contributions presented in the study are included in the article/Supplementary Material, further inquiries can be directed to the corresponding author.
